# The Effects of Two Novel Copper-Based Formulations on *Helicobacter pylori*

**DOI:** 10.3390/antibiotics2020265

**Published:** 2013-05-21

**Authors:** Ilaria M. Saracino, Cristina Zaccaro, Giobvanna Lo Re, Dino Vaira, John Holton

**Affiliations:** 1 First Medical Clinic, University of Bologna, Bologna 40121, Italy; E-Mails: iliaria.saracino@unibo.it (I.M.S.); crysette@hotmail.it (C.Z.); giovanna.lore@studio.unibo.it (G.L.R.); beradino.vaira@unibo.it (D.V.); 2 Department of Health & Social Sciences, University of Middlesex, NW4 4Bt London, UK

**Keywords:** bactericidal, copper, helicobacter, novel formulation

## Abstract

We investigated the effects of two novel copper-based inorganic formulations for their activity against 60 isolates of *Helicobacter pylori* (Hp). The two copper-based formulations were tested against three NCTC *Helicobacter pylori* isolates and 57 clinical strains isolated from the UK and Italy in time-kill assays. Both copper-based formulations were bio-cidal against all *Helicobacter pylori* strains tested reducing the viable count by 4–5 log within 2 h. These two copper-based anti-microbial agents deserve further study in relation to the treatment of *H. pylori*-related gastric disease.

## 1. Introduction

*Helicobacter pylori* is a gram negative, microaerophilic bacterium found principally in the stomach [1]. Infection with this organism is one of the most widespread human infections that affects up to 50% of the world’s population. Epidemiological studies have clearly demonstrated a major aetiological role for *Helicobacter pylori* in peptic ulcer disease, gastric MALT [mucosal associated lymphoid tissues] lymphoma, and distal gastric cancer [2,3,4,5]. Clarithromycin or metronidazole combined with amoxicillin and a proton pump inhibitor is the most frequently used regimen for first-line *Helicobacter pylori* therapy (standard triple therapy) in clinical practice in Europe as well as US [6]. This regimen is also advocated in the European Helicobacter Study Group (EHSG) Maastricht 2006 Guidelines. However, there are a number of problems with this approach. Currently, standard triple therapy fails to eradicate *Helicobacter pylori* infection in more than a third of patients [7]. Increasing primary resistance to clarithromycin or metronidazole, reported by different studies [8,9,10], is widely claimed as the most important factor reducing the efficacy of therapy. The prevalence of clarithromycin resistance varies between 6.1% to 14.5% in the USA and may be as high as 24% in some European countries, while the prevalence of metronidazole resistance ranges from 25% to 38% in Western countries, and up to 77% in some Asian countries. Amoxycillin resistance is rare. In a recent study, increasing rates of clarithromycin resistance (8.7%, 23.5% 26.7%, 34.5% were paralleled by falling eradication rates (90.6%, 80.2% 76% 74.8%) respectively [11]. 

A recently suggested first line treatment is sequential therapy, where the main aim is to overcome clarithromycin resistance [12]. Amoxycillin is given for five days followed by the addition of clarithromycin and a nitroimidazole for five days with a proton pump inhibitor given for the whole 10 days. Reported eradication rates are over 93.4% and are thus much better than current triple therapy results [13]. However, even this is only a Grade B success: a Grade A success is defined as >95% eradication [14,15,16]. It has also been suggested that sequential therapy should be a second-line therapy not a first line therapy. In areas where clarithromycin resistance is low, various modifications of sequential therapy have been used but none achieving Grade A eradication. 

The management of *Helicobacter pylori* infection after a failed course of initial treatment is considered more difficult, mainly due to selection of bacterial strains resistant to the antibiotics used in the primary eradication attempt. Thus, optimal therapy after first-line treatment failure remains controversial due to the lack of large randomized cohort studies and the lack of homogeneity of patients studied. In second or third line therapy, only a few antibiotics against *Helicobacter pylori* infection are suitable, such as bismuth compounds, levofloxacin, rifabutin or furazolidone, which are used in triple or quadruple regimes [6]. 

In summary, the treatment of *Helicobacter* associated disease is beset by the following problems: (1) Increasing antibiotic resistance; (2) Decreasing eradication rates from >90% to ~70%; (3) The need to take lots of tablets and therefore compliance problems leading to antibiotic resistance and failure of eradication; (4) The association of proton pump inhibitor use with *Clostridium difficile* colitis [17]. For these reason potential alternative treatments have been investigated such as inhibition of binding [18], photodynamic therapy [19], biocidal action of spices [20], anti-malarial drugs [21], essential oils [22] and other botanical extracts [23]. In this study, we have investigated the efficacy of novel highly reductive copper compounds for their bactericidal activity against *Helicobacter pylori*. 

## 2. Results and Discussion

### 2.1. Kill Curves of 5 NCTC/Clinical Isolates

**Figure 1:**
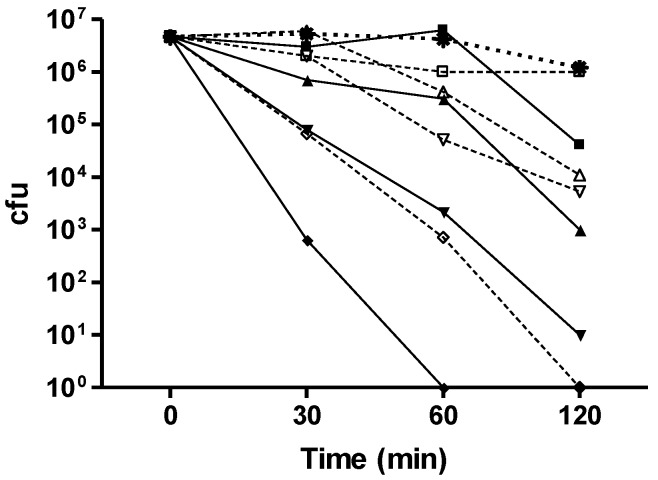
Time-kill curves with CuAL42 and CuPC33 on the antibiotic-sensitive *H. pylori* strain, J99.Star, control; closed square, CuAL42 0.5 mg/L; closed triangle, CuAL42 1 mg/L; closed upside-down triangle, CuAL42 5 mg/L; closed diamond, CuAL42 12 mg/L; open square, CuPC33 0.5 mg/L; open triangle, CuPC33 1 mg/L; open upside-down triangle, CuPC33 5 mg/L; open diamond, CuPC33 12 mg/L. The results shown are the means of triplicate observations. Error bars representing standard deviations (less than 10%) are not shown for reasons of clarity.

**Figure 2:**
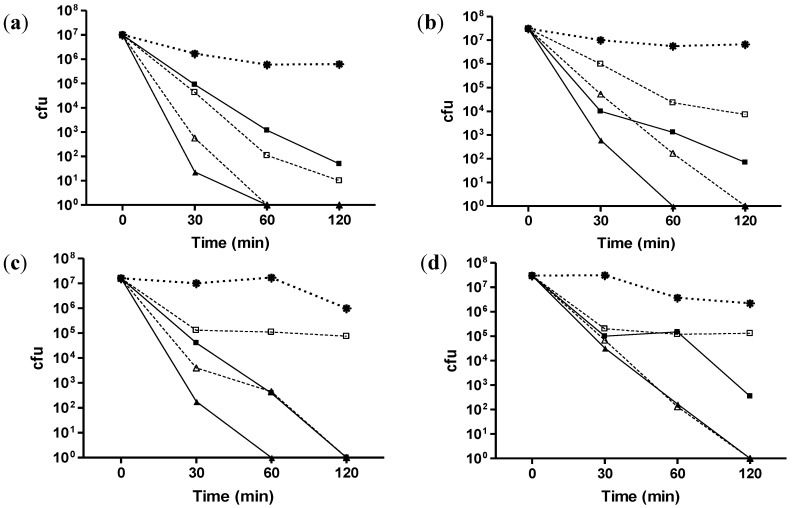
Time-kill curves with CuAL42 and CuPC33 on (**a**) CagA^−^; (**b**) CagA^+^; (**c**) metronidazole-resistant and (**d**) clarithromycin-resistant *H. pylori* strains. Star, control; closed square, CuAL42 5 mg/L; closed triangle, CuAL42 12 mg/L; open square, CuPC33 5 mg/L; open triangle, CuPC33 12 mg/L. The results shown are the means of triplicate observations. Error bars representing standard deviations (less than 10%) are not shown for reasons of clarity.

Both CuAL42 and CuPC33 had significant antimicrobial activity against *Helicobacter pylori*. A kill curve of ATCC strain J99 is illustrated in Figure 1. The kill curve of two NCTC isolates that were either Type II (cagA negative) or Type I (cagA positive) and 2 clinical isolates that were separately metronidazole resistant or clarithromycin resistant is illustrated in Figure 2 (a–d respectively) and demonstrated both compounds had similar activity and killed or reduced the number of bacteria by log >6 at 12 ppm, irrespective of virulence marker or antibiotic sensitivity. 

### 2.2. Activity of CuAL42 and CuPC33 against 57 Clinical Isolates

**Figure 3:**
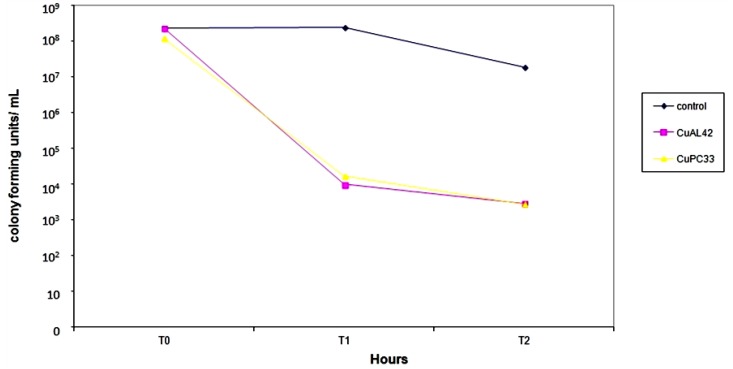
Mean Activity of CuAL42&CuPC33 against 53 clinical isolates of *H. pylori*. (AL42 = CuAL42; PC33 = CuPC33).

**Figure 4:**
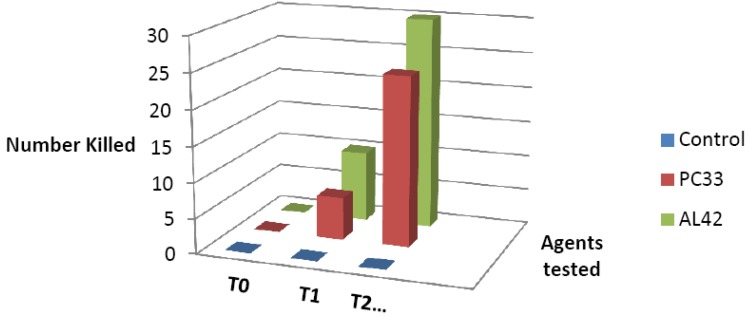
Number of *H. pylori* isolates killed at one hour (T1) and two hours (T2) by CuAL42 and CuPC33 (PC33 = CuPC33; AL42 = CuPC42).

The average reduction of viability for the 57 isolates is shown in Figure 3. The reduction in viability for both CuPC33 and CuAL42 was significant (*p* = 0.0001). The number of strains that were killed at 1 and 2 h is shown in Figure 4 where CuAL42 was slightly more active than CuPC33. 

The resistance rates of the clinical isolates were clarithromycin, (41%) metronidazole (58%) and levofloxacin (20%). Antibiotic resistance bore no relationship to the efficacy of the copper compound. 

### 2.3. Discussion

Previous studies have demonstrated these copper complexes killed *Acinetobacter baumanii* (ACCB) and methicillin resistant *Staphylococcus aureus* (MRSA) at a concentration of 150 ppm within 2 h, compared to equivalent concentrations of copper sulfate and the inorganic binders. At a concentration of 20 ppm the copper-based compounds also completely inhibited colony formation of ACCB and MRSA, but this required 16 hr of exposure [24]. More recently organo-metallic complexes including copper-fluoroquinolone have shown good antimicrobial activity against a small number of *Helicobacter pylori* strains [25]. In this study, we have analyzed the activity of CuPC33 and CuAL42 copper complexes against 60 stains of *Helicobacter pylori* and shown bactericidal activity against all isolates at a low concentration (12 ppm) within a period of 2 h. With increasing time, increasing numbers were killed. The specific advantage of the CuPC33 and CuAL42-complexes in this study, over the antibiotic-metal complexes [25], is the absence of any antibiotic. This suggests a lack of generating antibiotic resistant strains and possible not predisposing the patient to *Clostridium difficile* associated disease, although these effects would have to be confirmed by future work. 

Several mechanisms and target sites of action have been proposed for this very broad spectrum of biocidal activity of copper. Firstly, copper is a highly redox-active metallo-ion that promotes membrane lipid peroxidation, which in turn damages bacterial plasma membrane integrity [26]. Secondly, copper binds and disorders helical DNA by engaging this structure at two different binding sites, explaining its activity against a wide range of viruses [27]. Thirdly, copper facilitates free radical–mediated degradation of proteins, which includes on the one hand oxidation of specific amino acid residues, and on the other degradation of sulfydryl moieties [28]. The mechanism by which the inorganic binders so effectively enhance copper’s biocidal activity is currently under investigation. Preliminary experiments using scanning electron microscopy indicate that exposure of ACCB to CuAL42 (150 ppm) for 20 min causes extensive membrane “blebbing” [24]. 

Previous work has indicated a lack of cytotoxicity for eukaryotic cells indicating a selective toxicity for bacterial cells at the concentrations used [24,29]. Eukaryotic cells have a recognized ability to withstand copper toxicity by well characterized, often highly complex mechanisms of intracellular protein-dependent coordination, chelation, and transport, and to well characterized extracellular binding pathways in multicellular higher organisms [30]. 

Finally, the use of copper complex for *Helicobacter pylori*-associated peptic ulcer disease has one further possible advantage: that of enhancing ulcer healing. One of the earliest events in ulcer healing is rapid epithelial cell migration leading to reconstitution of the epithelium [31]. This is followed by cell proliferation, which restores the mucosal epithelium to its normal thickness. Angiogenesis is essential for providing blood flow to the re-establishing epithelium and for the creation of new glands [32]. In animal models, it has been shown that the ulcer repair process can be accelerated by the administration of epidermal growth factor (EGF) *via* an effect on both angiogenesis and re-epithelialization [33]. Copper is recognized to have angiogenic properties by stimulating VEGF and hypoxia-inducible factor-1 alpha [34,35] and it is not unreasonable to suppose that these copper complexes may have a similar action, although experimental data is currently lacking. 

## 3. Experimental

### 3.1. Materials

Bacteria: 3 NCTC cultures—NCTC11637, NCTC 12908, ACTC J99 and 57 clinical isolates, 43 from Italy and 14 from the UK were used. Copper compounds: The two copper-based formulations—CuPC33 and CuAL42—were provided by Remedy Research Ltd. These formulations are composed of copper sulfate and two inorganic compounds that form the metallo-ion binders. The binders comprise an ammonium salt and an inorganic acid—ammonium phosphate and phosphoric acid in PC33 and ammonium sulfate and sulfuric acid in AL42. The concentration of elemental copper in each stock solution was 30.43 g/L.

### 3.2. Determination of Antibiotic Sensitivity

Clarithromycin, levofloxacin and metronidazole sensitivity was determined on all isolates using European Guidelines [36]. Briefly, testing was performed on Muller Hinton agar containing 10% horse blood with an inoculum of 0.5 × 10^9^. Plates were incubated at 37 °C in a micro-aerobic atmosphere (CampyGen Oxoid, Basingstoke, UK) for 3 days. Clarithromycin and levofloxacin resistance was determined by E test (AB Biodisc Solna Sweden) with resistance recorded as > or equal to 1.0 g/mL for both and resistance to metronidazole determined by breakpoint agar streak with growth at 8.0 g/mL denoting resistance. 

### 3.3. Determination of Activity of CuAL42 and CuPC33

Cag A positive (NCTC11637); CagA negative (NCTC 12908 and ACTC J99) and 57 clinical isolates were tested. For 5 of the isolates (3 Type strains and 2 clinical isolates) a time-kill curve was performed with an inoculum of 10^7−8^ cfu/mL in sterile water at differing concentrations (0.5, 1.0, 5.0, and 12 ppm) of the two copper compounds CuAL42 and CuPC33 against a control containing either sterile water, copper sulfate or binders, for 30, 60 and 120 minutes. At each time point, samples were withdrawn, decimal diluted into ¼ strength Ringers lactate, plated onto blood agar (Oxoid Ltd.) and incubated. Subsequently all the isolates were tested by agar incorporation using Muller Hinton agar with 10% horse blood at the same inoculum against 12 ppm for 60 and 120 mins. The plates were incubated for 5 days at 37 °C in an atmosphere generated by CampyGen (Oxoid Ltd.). 

### 3.4. Statistics

A student’s *t*-test was performed comparing the mean viable count of the control against the test for both agents at T1 and T2. 

## 4. Conclusions

Our conclusions are that these reductive copper compounds deserve further studies in relation to the mechanism of killing, resistance rate and effects on ulcer healing separate from the bactericidal activity reported here. 

## References

[1] Warren J.R., Marshall B.J. (1983). Unidentified curved bacilli on gastric epithelium in active chronic gastritis. Lancet.

[2] Sung J.J., Kuipers E.J., El-Serag H.B. (2009). The global incidence and prevalence of PUD. Aliment. Pharmacol. Ther..

[3] Atherton J.C. (1997). The clinical relevance of strain types of *Helicobacter pylori*. Gut.

[4] IARC Working Group on the Evaluation of Carcinogenic Risks to Humans (1994). Schistosomes, Liver Flukes and *Helicobacter pylori*.

[5] Santacroce L., Cagiano R., del Prete D., Bottalico L., Sabatini R., Carlaio R.G., Prejbeanu R., vermesan H., Dragulescu S.I., Vermesan D. (2008). *Helicobacter pylori* infection and gastric MALToma: An up to date and therapeutic highlight. Clin. Ter..

[6] O’Connor A., Gisbert J.P., McNamara D., O’Morain C.A. (2010). Treatment of *Helicobacter pylori* infection 2010. Helicobacter.

[7] Megraud F. (2004). *H pylori* antibiotic resistance: Prevalence, importance and advances in testing. Gut.

[8] Koletzko S., Richy F., Bontems P., Crone J., Lalach N., Monteiro M.L., Gottrand F., Celinska-Cedro D., Roma-Giannikou E., Oderda G. (2006). Prospective multicentre study on antibiotic resistance of *Helicobacter pylori* strains obtained from children living in Europe. Gut.

[9] Ramond J., Lamarque D., Kalach N., Chaussade S., Burucoa C. (2010). High levels of antimicrobial resistance in French *Helicobacter pylori* isolates. Helicobacter.

[10] Agudo S., Alarcon T., Cibrelus L., Urruzuno P., Martinez M.J., Lopez-Brea M. (2009). High percentage of clarithromycin and metronidazole resistance in *Helicobacter pylori* clinical isolates obtained from Spanish children. Rev. Esp. Quimioter..

[11] Sasaki M., Ogasawara N., Utsumi K., Kawamura N., Kamiya T., Kataoka H., Tanida S., Mizoshita T., Kasugai K., Joh T. (2010). Changes in 12 year first line eradication rate of *Helicobacter pylori* based on triple therapy with proton pump inhibitor, amoxicillin and clarithromycin. J. Clin. Biochem. Nutr..

[12] Gisbert J.P., Calvet X., O’Connor A., Megraud F., O’Morain C. (2010). A Sequential therapy for *Helicobacter pylori* eradication: A critical review. J. Clin. Gastroenterol..

[13] Jafri N.S., Hornung C.A., Howden C.W. (2008). Meta analysis: Sequential therapy appears superior to standard therapy for *Helicobacter pylori* infection in patients naïve to treatment. Ann. Int. Med..

[14] Graham D.Y., Lu H., Yamaoka Y. (2007). A report card to grade Helicobacter therapy. Helicobacter.

[15] Graham D.Y., Lu H., Yamaoka Y. (2008). Therapy for *Helicobacter pylori* infection can be improved: Sequential therapy and beyond. Drugs.

[16] Graham D.Y. (2009). Efficient identification and evaluation of effective *Helicobacter pylori* therapies. Clin. Gastroenterol. Hepatol..

[17] Rotramel A., Poritz L.S., Messaris E., Berg A., Stewart D.B. (2012). PPI therapy and albumen are better predictors of recurrent *Clostridium difficile* colitis than choice of antibiotics. J. Gastrointest. Surg..

[18] Younson J., O’Mahony R., Liu H., Basset C., Grant S., Campion C., Jennings L., Vaira D., Kelly C.G., Roitt I.M. (2009). Human domain antibody and Lewis^b^-glycoconjugate that inhibit binding of *Helicobacter pylori* to Lewis^b^ receptor and adhesion to human gastric epithelium. J. Infect. Dis..

[19] Bedwell J., Holton J., Vaira D., MacRobert A.J., Bown S.G. (1990). *In vitro* killing of *Helicobacter pylori* with photodynamic therapy. Lancet.

[20] O’Mahony R., Al-Khtheeri H., Weerasekera D., Fernando N., Vaira D., Holton J., Basset C. (2005). Bactericidal and Anti-adhesive properties of culinary and medicinal plants against *Helicobacter pylori*. World J. Gastroenterol..

[21] Boschian A., Jensen C.S., Rasmussen L. (2010). Antibiotic effect of anti-malarials on *H. pylori* infection *in vivo*. Helicobacter.

[22] Menghini L., Leporini L., Tirillini B., Epifano F., Genovese S. (2010). Chemical composition and inhibitory activity against *Helicobacter pylori* of the essential oil of *Apium nodiflorum* (Apiaceae). J. Med. Food.

[23] Lawal T.O., Adeniyi B.A., Moody J.O., Mahady G.B. (2010). Combination studies of *Eucalyptus torelliana* (F MUELL) leaf extract and clarithromycin on *H. pylori*. Helicobacter.

[24] Gant V.A., Wren M.W., Rollins M.S., Jeanes A., Hickok S.S., Hall T.J. (2007). Three novel highly charged copper based biocides: Safety and efficacy against healthcare associated organisms. J. Antimicrob. Chemother..

[25] Shaikh A.R., Yadav M., Giridhar R. (2010). Antibiotic metal complexes—An approach for *Helicobacter* therapy. Helicobacter.

[26] Cervantes C., Gutierrez-Corona F. (1994). Copper resistance mechanisms in bacteria and fungi. FEMS Microbiol. Rev..

[27] Sagripanti J.L., Routson L.B., Lytle C.D. (1993). Virus inactivation by copper or iron ions alone and in the presence of peroxide. Appl. Environ. Microbiol..

[28] Kim J.H., Cho H., Ryu S.E., Choi M.U. (2000). Effects of metal ions on the activity of protein tyrosine phosphatise VHR: Highly potent and reversible oxidative inactivation by Cu^2+^ ion. Arch. Biochem. Biophys..

[29] Hall T.J., Wren M.W., Jeanes A., Gant V.A. (2009). A comparisonof the antibacterial efficacy and cytotoxicity to cultured human skin cells of 7 commercial hand rubs and Xgel, a new copper-based biocidal hand rub. Am. J. Infect. Control..

[30] Koch K.A., Pena O., Thiele D.J. (1997). Copper binding motifs in catalysis, transport, detoxification and signalling. Chem. Biol..

[31] Silen W., Ito S. (1985). Mechanisms for rapid re-epithelialization of the gastric mucosal surface. Annu. Rev. Physiol..

[32] Wallace J.L. (2001). Mechanisms of protection and healing: Current knowledge and future research. Am. J. Med..

[33] Konturek S.J., Dembinski A., Warzecha Z., Brzozoski T., Gregory H. (1992). Role of epidermal growth factor in healing of chronic gastroduodenal ulcers in rats. Gastroenterology.

[34] Borkow G., Gabbay J., Dardik R., Eidelman A.I., Lavie Y., Grunfeld Y., Ikher S., Huszar M., Zatcoff R.C., Marikovsky M. (2010). Molecular mechanisms of enhanced wound healing by copper-oxide impregnated dressings. Wound Repair Regen..

[35] D’Andrea L.D., Romanelli A., Di Stasi R., Pedone C. (2010). Bioinorganic aspects of angiogenesis. Dalton Trans..

[36] Megraud F., Lehors P. (2007). *Helicobacter pylori* detection and antimicrobial susceptibility testing. Clin. Microbiol. Rev..

